# Patients’ willingness to take separate component antiretroviral therapy regimens for HIV in the Netherlands

**DOI:** 10.7448/IAS.17.4.19536

**Published:** 2014-11-02

**Authors:** Esther Engelhard, Colette Smith, Sigrid Vervoort, Frank Kroon, Kees Brinkman, Pythia Nieuwkerk, Peter Reiss, Suzanne Geerlings

**Affiliations:** 1 Division of Infectious Diseases, Academic Medical Center, University of Amsterdam, Amsterdam, Netherlands; 2 Stichting HIV Monitoring, Amsterdam, Netherlands; 3 Department of Internal Medicine and Infectious, University Medical Center, Utrecht, Netherlands; 4 Department of Infectious Diseases, Leiden University Medical Center, Leiden, Netherlands; 5 Department of Internal Medicine, Onze Lieve Vrouwe Gasthuis, Amsterdam, Netherlands; 6 Department of Medical Psychology, Academic Medical Center, University of Amsterdam, Amsterdam, Netherlands; 7 AIGHD, Academic Medical Center, University of Amsterdam, Amsterdam, Netherlands

## Abstract

**Introduction:**

The costs of combination antiretroviral therapy (cART) consisting of separate, particularly generic, components are generally much lower than of a single tablet regimen (STR) including the same active ingredients. Our aim was to evaluate whether patients in care in the Netherlands would be willing to take separate component regimens (SCR) instead of an STR and to examine whether willingness was associated with particular patient characteristics.

**Materials and Methods:**

Data from the HIV Monitoring Foundation of all adult HIV-1-infected patients in care taking cART>6 months were used to randomly select 1000 patients. As part of a questionnaire developed for a study assessing patient experience, patients were asked whether they were willing to take an SCR instead of an STR. Logistic regression was used to examine associations between age, gender, region of origin, mode of HIV transmission, socioeconomic status, duration of cART and answering “yes” to the question versus “maybe” or “no.” Variables with p<0.1 in the univariate analysis were entered in a multivariate model.

**Results:**

Of the 300 patients who completed the questionnaire, 49% answered “yes,” 24% “maybe” and 27% “no” to the question whether they would be willing to use a SCR. Reasons for answering “no” included difficulties swallowing pills, convenience of STR (especially when travelling/at work), and concerns about side effects. Respondents who answered “maybe” often indicated that they preferred STRs, emphasized the importance of taking the pills once daily, and pointed out that efficacy/safety of an SCR should not be less. Having to pay for medication was reported as a reason to consider switching to an SCR. In the multivariate analysis, respondents who were born outside the Netherlands were less likely; and those with cART use ≥15 yrs were more likely to answer “yes” (Table 1).

**Conclusions:**

Half of the respondents were willing to take SCRs instead of an STR. The likelihood of accepting to switch to SCR seems less for migrants and for those who have commenced treatment more recently. Duration of cART use and region of origin may therefore be factors to take into account when considering to prescribe SCR. Future studies should investigate whether an expressed willingness to switch will translate into maintained high levels of adherence and viral suppression.

**Table 1:** Adjusted^a^ odds ratios (OR), 95% confidence intervals (95% CI) and *p*-values for respondents (n=300) reporting to be willing to use a separate component regimen

Characteristics	n (%)	OR (95%): “yes” vs. “maybe/no”	p
Region of origin			
Netherlands	234 (78)	Ref	
Other	66 (22)	0.32 (0.16–0.64)	0.001
Duration of cART use (yrs)			
< 5	78 (26)	Ref	
5–10	88 (29)	1.73 (0.88–3.40)	0.110
10–15	66 (22)	0.69 (0.33–1.46)	0.334
≥ 15	68 (23)	3.18 (1.49–6.79)	0.003

a Adjusted for age, gender, mode of transmission and socioeconomic status.

